# A simple and robust reporter-based framework for deep functional characterization of PPARγ mutants

**DOI:** 10.1210/endocr/bqag024

**Published:** 2026-03-06

**Authors:** Rosalie Baak, Denise Westland, Eline de Lange, Rene Houtman, Eric Kalkhoven

**Affiliations:** Center for Molecular Medicine, University Medical Center Utrecht, Utrecht University, 3584 CG Utrecht, the Netherlands; Center for Molecular Medicine, University Medical Center Utrecht, Utrecht University, 3584 CG Utrecht, the Netherlands; Center for Molecular Medicine, University Medical Center Utrecht, Utrecht University, 3584 CG Utrecht, the Netherlands; Precision Medicine Lab, Antoni van Leeuwenhoek Building, 5349AE Oss, the Netherlands; Center for Molecular Medicine, University Medical Center Utrecht, Utrecht University, 3584 CG Utrecht, the Netherlands

**Keywords:** nuclear receptors, PPARγ, natural missense mutants, transcriptional regulation, coregulator interaction

## Abstract

Missense mutations in nuclear receptor (NR) transcription factors cause a number of genetic disorders, including *PPARG* mutations that result in familial partial lipodystrophy type 3 (FPLD3). Experimental assessment is essential to establish a newly identified mutation as disease-causing, as accurately predicting the effect of a new mutation in silico remains challenging due to the multifunctional and modular nature of these proteins. However, deep structure–function characterization often requires specialized and technically demanding approaches, which may not be readily available. Therefore, we established a simple and robust experimental framework based on 4 complementary reporter assays that independently assess (1) ability of the full-length receptor to activate transcription; (2) integrity of the ligand-binding domain; (3) heterodimerization potential; and (4) DNA-binding capacity. As a proof of concept, we analyzed 3 uncharacterized FPLD3-associated loss-of-function variants and 2 bladder cancer–associated gain-of-function variants. Together, the 4 complementary assays showed unique functional phenotypes for all 5 mutants that were further supported by coregulator profiling. We therefore conclude that this framework provides a simple and robust first-line approach to identify functional alterations in peroxisome proliferator–activated receptor γ mutants with mechanistic resolution. This framework is broadly applicable across NRs and offers a scalable path to systematic variant interpretation both in research and clinical contexts.

Nuclear receptors (NRs), a class of 48 transcription factors in humans, are involved in a plethora of fundamental cellular processes, including proliferation, differentiation, homeostasis, and metabolism and are consequently also implicated in human disease (eg, cancer, metabolic diseases) (1, 2). Genetic variants in NR genes have been linked to several human diseases, including several “classic” hormone resistance syndromes (3), and provide a powerful tool to unravel not only the physiological functions of NRs but also the molecular mechanisms underlying their role in gene regulation. NRs contain an N-terminal activation function-1 (AF-1) domain, a DNA-binding domain (DBD) that specifically recognizes and binds response elements in enhancer and promoter regions in the genome, and a C-terminal ligand-binding domain (LBD) with an activation function-2 (AF-2) surface. Ligand binding provides a molecular switch for gene activation, particularly for type II (nonsteroid) NRs; corepressors, such as silencing mediator for retinoic acid receptor (SMRT) and NR corepressor (NcoR), are bound to the LBD prior to ligand binding through their LXXXIXXXL motifs, and on ligand binding helix 12 (H12) undergoes a conformational shift in H12, resulting in release of corepressors. For both type I (steroid) and type II NRs, this ligand-dependent conformational change generates a docking surface for the recruitment of coactivators via their LXXLL motifs, including steroid receptor coactivator-1 (SRC1) and CREB-binding protein (CBP) (1, 2, 4). A clear example of an NR directly linked to homeostasis and disease is peroxisome proliferator–activated receptor γ (PPARγ). PPARγ heterodimerizes with retinoid X receptor (RXR)α and regulates gene programs involved in adipocyte differentiation and metabolism (5‐7). The role of PPARγ in adipose tissue is underscored by loss-of-function (LOF) mutations that cause familial partial lipodystrophy type 3 (FPLD3; OMIM 604367), a syndrome characterized by severe metabolic consequences such as insulin resistance, type 2 diabetes mellitus, and dyslipidemia (6, 7). In contrast, gain-of-function (GOF) mutations of PPARγ have been reported in luminal muscle-invasive bladder cancer, where they drive aberrant PPARγ signaling (8‐11) To date, multiple computational methods have been developed for genetic variant effect prediction (12‐20), including those that leverage protein sequence and/or structure using AlphaFold (21‐23). Unfortunately, distinguishing disease-causing genetic variants from rare benign variants and assigning functional consequences solely based on in silico predictions remain a challenge given the complex nature of PPARγ signaling—and NR signaling in general—with single missense mutations affecting multiple mechanisms simultaneously, either directly or through allosteric effects, as recently illustrated by specific PPARγ mutants (24). Therefore, experimental validation to determine the pathogenicity and mechanistic basis of missense variants identified in individuals is still needed for accurate clinical diagnosis (23). However, deep structure–function characterization may require specialized, resource-intensive assays, such as x-ray crystallography, hydrogen/deuterium exchange mass spectrometry, radioactive ligand-binding experiments, and electrophoretic mobility shift assays (24‐26), limiting rapid and systematic analysis of missense variants. Therefore, new first-line screening methods to characterize potential pathogenic missense mutations of PPARγ are needed. Here we present a reporter-based framework that provides an effective first-line approach for assessing functional effect of missense variants and mapping potential defects to certain domain(s). As proof of principle, we applied our approach to 3 FPLD3-associated LOF mutants and 2 bladder cancer–associated GOF mutants, providing robust characterization of distinct functional phenotypes, which can be easily adapted for other NRs.

## Materials and methods

### Cell culture

The human osteosarcoma cell line U2OS and the human embryonic kidney cell line HEK293T were cultured in Dulbecco's modified Eagle's medium (DMEM; Gibco) 4.5-g/L D-glucose supplemented with 10% fetal bovine serum (Gibco), and 100-units penicillin/mL and 100-μg/mL streptomycin (Invitrogen).

### Luciferase-based reporter assays

The pGL3-*mLpl*-PPRE-Luc2, pGL3-*mCidec*-PPRE-Luc2, and pGL3-*mSynthetic*-PPRE-Luc2 reporter constructs have previously been described (24). The *Lpl* reporter construct was then used as a template to generate the *Angptl4* reporter using the QuickChange mutagenesis kit (Agilent) following instructions provided by the manufacturer. PPRE sequences are provided in Supplementary Table S1 (27). The reporter construct 5xGal4-E1BTATA-pGL3 has previously been described (28).

pCDNA3.1 expression vectors for hPPARγ2 and hRXRα and Gal4DBD-hPPARγ-LBD have been described previously (29). The pcDNA3.1-VP16-HA-hPPARγ2 expression vector was generated by inserting the VP16 transactivation domain (from the Herpes simplex virus protein vmw65) N-terminally fused to HA-tagged hPPARγ2 into the pCDNA3.1 backbone using the In-Fusion cloning kit (Takara) following instructions provided by the manufacturer. All mutant variants were generated using the QuickChange mutagenesis kit (Agilent) following the manufacturer's instructions. Primer sequences are provided in Supplementary Table S2 (27).

Luciferase reporter assays were performed exactly as described previously (24). Results are averages of at least 3 independent experiments assayed in duplicate ± SEM. To compare 3 or more groups, a 2-way analysis of variance was performed with a Dunnet's multiple comparison test to compare the mean of each group with that of every other group. A statistically significant difference was defined as a *P* value less than .05.

### Western blot analysis

Protein expression by Western blot was performed exactly as described previously (24). The following primary antibodies were used for protein detection: anti-PPARγ (sc-7196; RRID: AB_654710), anti-Gal4 DBD (sc-510; RRID: AB_627655), anti-RXRα (sc-553; RRID: AB_2184874), anti-tubulin (Sigma–Aldrich T9026; RRID: AB_477593), anti-HA (ab9110; RRID: AB_307019), and anti-LgBiT (N7100; RRID: AB_3246441). All antibodies were used at a dilution of 1:1000, except for the anti-Gal4 DBD antibody, which was diluted 1:500.

### Protein complementation assays

PPARγ2-RXRα heterodimerization was analyzed in live HEK293T cells with the NanoBiT PPI System (Promega) exactly as described previously (24). The results are averages of at least 3 independent experiments assayed in triplicate ± SEM. To compare 3 or more groups, 2-way analysis of variance was performed with a Dunnet's multiple comparison test to compare the mean of each group with that of every other group. A statistically significant difference was defined as a *P* value less than .05.

### Nuclear receptor cofactor profiling

Ligand-induced interaction of wild-type (WT) PPARγ and mutants was assessed using the NR activity profiling assay as previously described (30). HEK293T cells were transfected with Gal4DBD-hPPARγ-AF2 WT or mutant versions using branched polyethylenimine (Sigma-Aldrich, 408 719) in 15-cm plates. Cells were scraped in phosphate-buffered saline and snap-frozen and stored at −80 °C, 48 hours post transfection. Cell lysates were prepared and a set of immobilized peptides representing coregulator-derived NR binding motifs was incubated with a 25-μL reaction mixture containing 10-μL cell lysate, Tris-buffer saline (TBS: 0.05 M Tris and 0.15 M NaCl, pH 7.6) with 0.05% Tween-20, 0.2% bovine serum albumin, 50-μM DTT, 1-μM rosiglitazone, and Gal4 antibody (Santa Cruz, sc-510; RRID: AB_627655). Incubation was performed for 40 minutes at 20 °C, followed by washing to remove unbound receptor/fluorophore and generation of a tiff image of each array. To compare clones, raw binding values were normalized in silico based on PPARγ protein input as quantified by Western blot using Gal4 detection.

## Results

### A systematic reporter-based framework to functionally characterize peroxisome proliferator–activated receptor γ mutants

**Figure 1:**
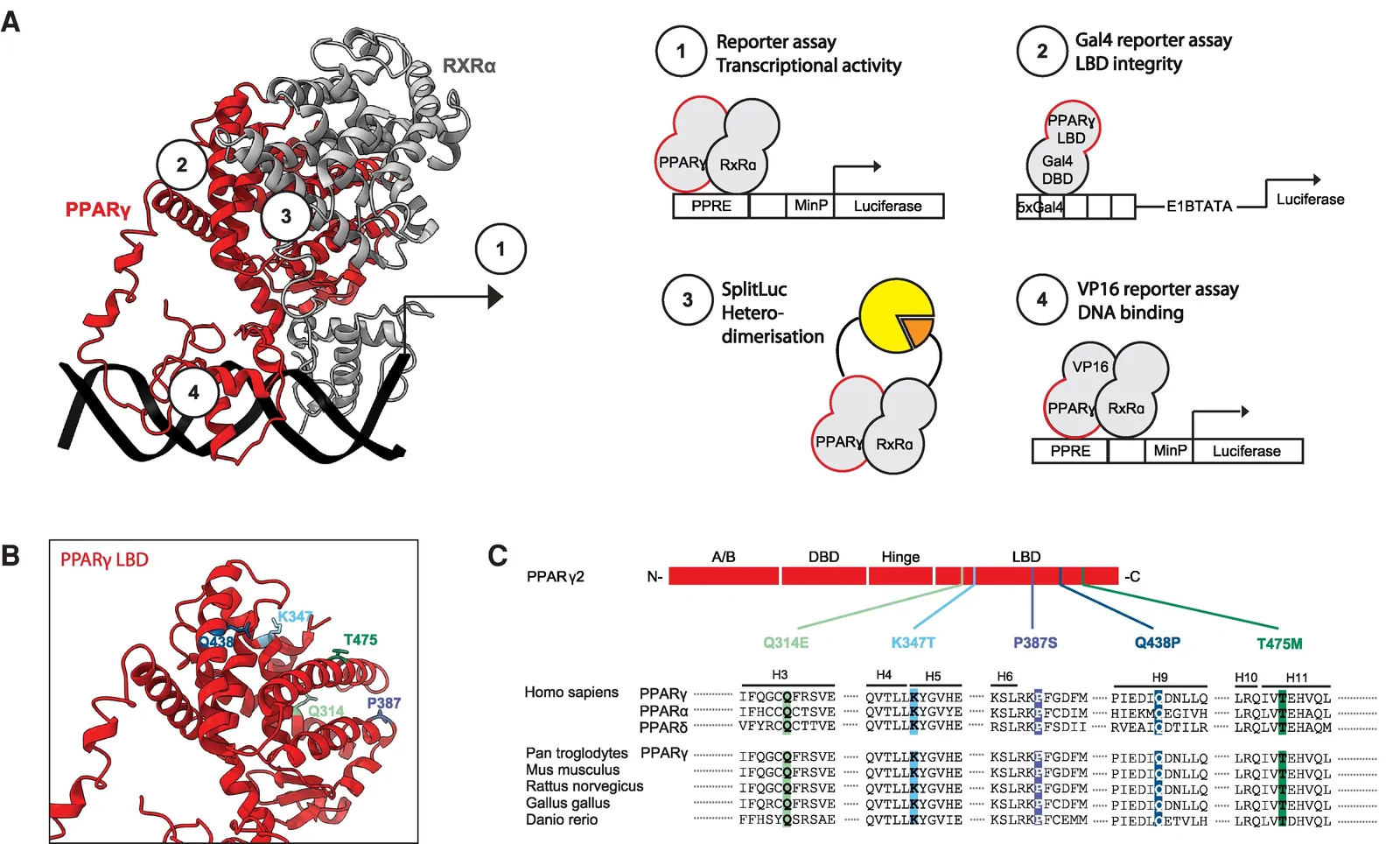
Schematic overview of systematic method to characterize the molecular dysregulation of nuclear receptor (NR) mutants. A, Crystal structure of PPARγ2:RXRa heterodimer bound to DNA (PPARγ in red; RXRa in gray; PDB ID: 3DZY) with numerical annotations indicating the 4 functional aspects of peroxisome proliferator–activated receptor γ (PPARγ) activity evaluated by our method: (1) the ability of the full-length receptor to activate transcription; (2) the integrity of the ligand-binding domain (LBD); (3) the heterodimerization potential; and (4) the DNA binding capacity. B, Zoom-in of PPARγ2-LBD with the 5 mutations Q314E, K347T, P387S, Q438P, and T475M annotated in color. C, Top: Schematic representation of domains in PPARγ2; N-terminal A/B-domain, DNA-binding domain (DBD), hinge region, and LBD and indicated positions of the 5 mutations. Bottom: Alignment of the amino acid sequence surrounding PPARγ2 Q314, K347, P387, Q438, and T475 between human PPAR subtypes and PPARγ across different species. Residue positions of the 5 mutations are highlighted in color.

To enable the deep characterization of PPARγ mutants, we established a simple and robust reporter-based framework that examines (1) the ability of the full-length receptor to activate transcription; (2) the integrity of the LBD; (3) its heterodimerization potential; and (4) its DNA-binding capacity. First, transcriptional activity is assessed using a conventional reporter assay (Fig. 1A.1). As we have recently shown that the behavior of some natural mutants critically depends on the peroxisome proliferator response element (PPRE) sequence (24), reporters harboring different PPREs are included. Second, we reasoned that for any LBD mutant, it is essential to verify whether the structural integrity of the LBD itself is affected. For this, mutant LBDs are fused to a Gal4 DBD and tested in a Gal4 reporter assay (Fig. 1A.2). Third, the effect of a mutant on the heterodimerization with RXRα is assessed by a split-luciferase protein complementation assay (Fig. 1A.3) (24). Last, the effect of a mutant on DNA binding is assessed by fusing the potent transactivation domain VP16 to full-length PPARγ (25). In this setup, transcriptional activation reflects the mutant's ability to bind DNA independent of its intrinsic transactivation potential (Fig. 1A.4).

### Characterization of familial partial lipodystrophy type 3–associated loss-of-function peroxisome proliferator–activated receptor γ mutants

**Figure 2:**
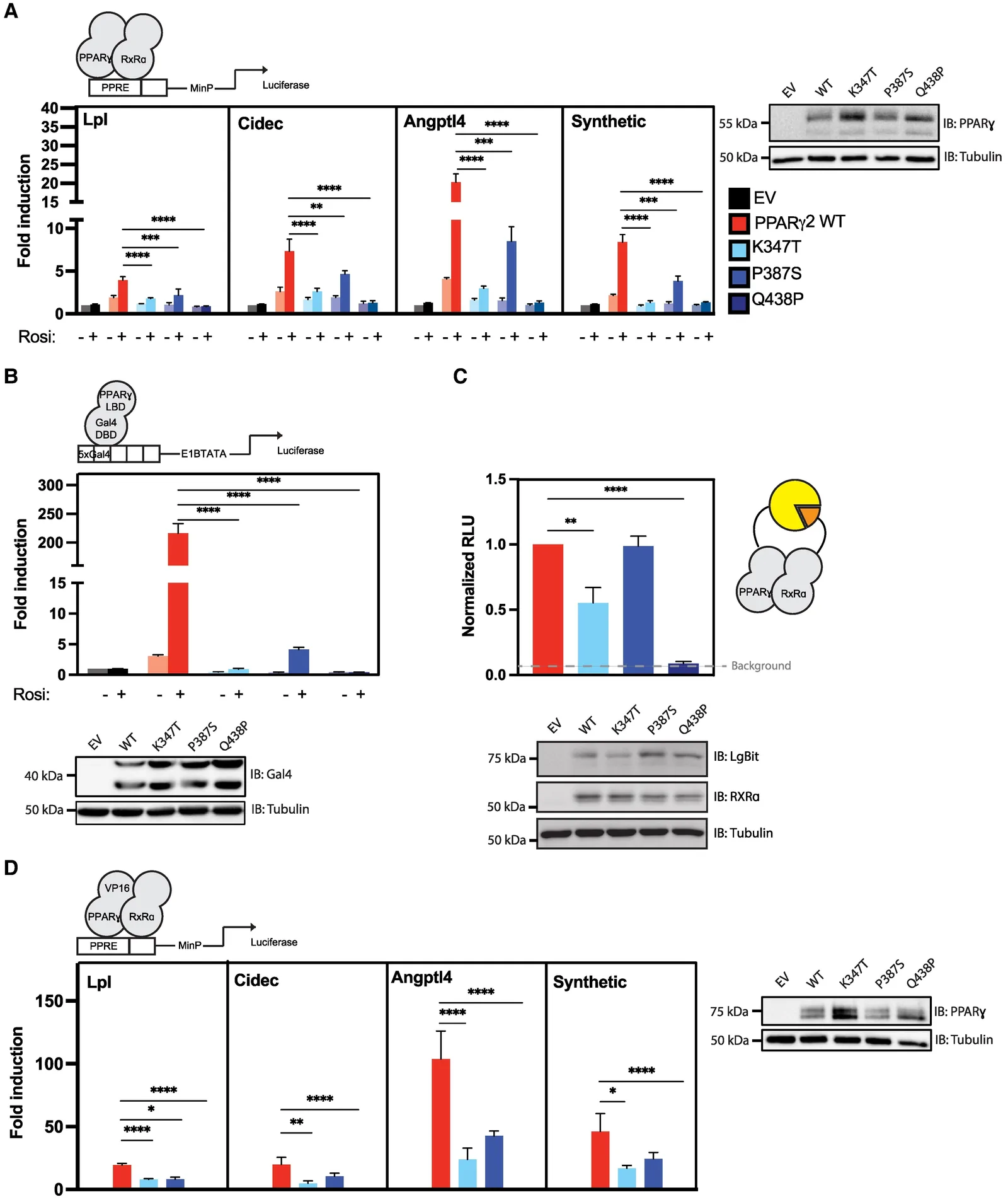
Characterization of lipodystrophy-associated loss-of-function (LOF) mutations. U2OS cells were transiently cotransfected with expression vectors encoding A, PPARγ-WT or LOF mutants, or B, Gal4DBD-PPARγ-WT or Gal4DBD-LOF-mutants and different reporter constructs or a Gal4 reporter construct as indicated, in the absence or presence of 1-µM rosiglitazone. Activation of the reporter is expressed as fold induction over that with empty vector (control). C, HEK293T cells were cotransfected with expression vectors encoding PPARγ2-LgBiT or LOF mutants, and smBiT-hRxRα. Luciferase activity is expressed relative to PPARγ2-WT. D, U2OS cells were transiently cotransfected with expression vectors encoding VP16-PPARγ-WT or VP16-LOF mutants and different reporter constructs in the absence of ligand. Activation of the reporter is expressed as fold induction over that with empty vector (control). Protein expression levels were verified by Western blot for all assays with annotated antibodies. All data are presented as mean values + SEM with n = 4 biologically independent experiments. Mutant results were compared to wild-type (WT) with a Dunnett's multiple comparison test: *.01 < *P* < .05; **.001 < *P* < .01; ***.0001 < *P* < .001; ****P < .0001.

FDLD3-associated PPARγ mutations provide an excellent test case for the experimental framework described earlier, as they invariably cause LOF, but the detailed molecular mechanisms may differ and more than one function can be affected (7). Of special interest are LBD mutations, as not only ligand binding and subsequent cofactor binding, but also RXR dimerization and thereby DNA binding critically depend on this region of the protein. We selected 3 largely uncharacterized FPLD3-associated mutations of highly conserved residues in the LBD of PPARγ (Fig. 1B and 1C): K347T, P387S (31, 32), and Q438P (32). While no functional data regarding the K347T and Q438P mutants are available, the P387S mutant was shown to have reduced transcriptional activity but further molecular details were not reported (31). We first investigated the effect of these 3 mutations on the ability of PPARγ to activate transcription, comparing reporter constructs that contain natural PPREs located in the enhancers and/or promoters of the lipoprotein lipase (*Lpl*) gene, cell death–inducing DFFA like effector C gene (*Cidec*), or the angiopoietin-related protein 4 (*Angptl4*) gene, as well as a synthetic perfect palindromic PPRE (24). All 3 mutants showed significantly reduced ability to activate the 4 reporter constructs compared to WT (Fig. 2A) at comparable levels of expression. Notably, P387S showed the mildest impairment. Second, we investigated the effect of the 3 mutations on LBD integrity. All 3 mutants exhibited greatly reduced ability to activate transcription at comparable levels of expression, indicating disruption of the LBD's structural integrity (Fig. 2B). Third, we assessed the ability of the mutants to heterodimerize with RXRα (24). Heterodimerization of the P387S mutant was comparable to WT protein, while the K347T and Q438P mutants showed partial and complete loss of heterodimerization, respectively (Fig. 2C). Last, we evaluated the ability of the mutants to bind to the aforementioned PPREs. The K347T and Q438P mutants showed substantially reduced activity on all 4 reporters, indicating destabilized DNA-binding (Fig. 2D). These findings are consistent with the observation that these mutants exhibit impaired heterodimerization (see Fig. 2C), which is a prerequisite for effective DNA binding (33). The P387S mutant, which maintained heterodimerization, exhibited destabilized DNA binding as well, in particular on the *Lpl* reporter (Fig. 2D). In summary, these findings show that our reporter-based framework can discriminate between functional PPARγ defects present in FPLD3 patients: The K347T and Q438P mutants display partial and dramatic loss of heterodimerization capacity and subsequent DNA binding, respectively, while the defect of the P387S mutant most likely resides on an additional functional level since heterodimerization remained intact (discussed next).

### Characterization of bladder cancer–associated gain-of-function peroxisome proliferator–activated receptor γ mutants

**Figure 3:**
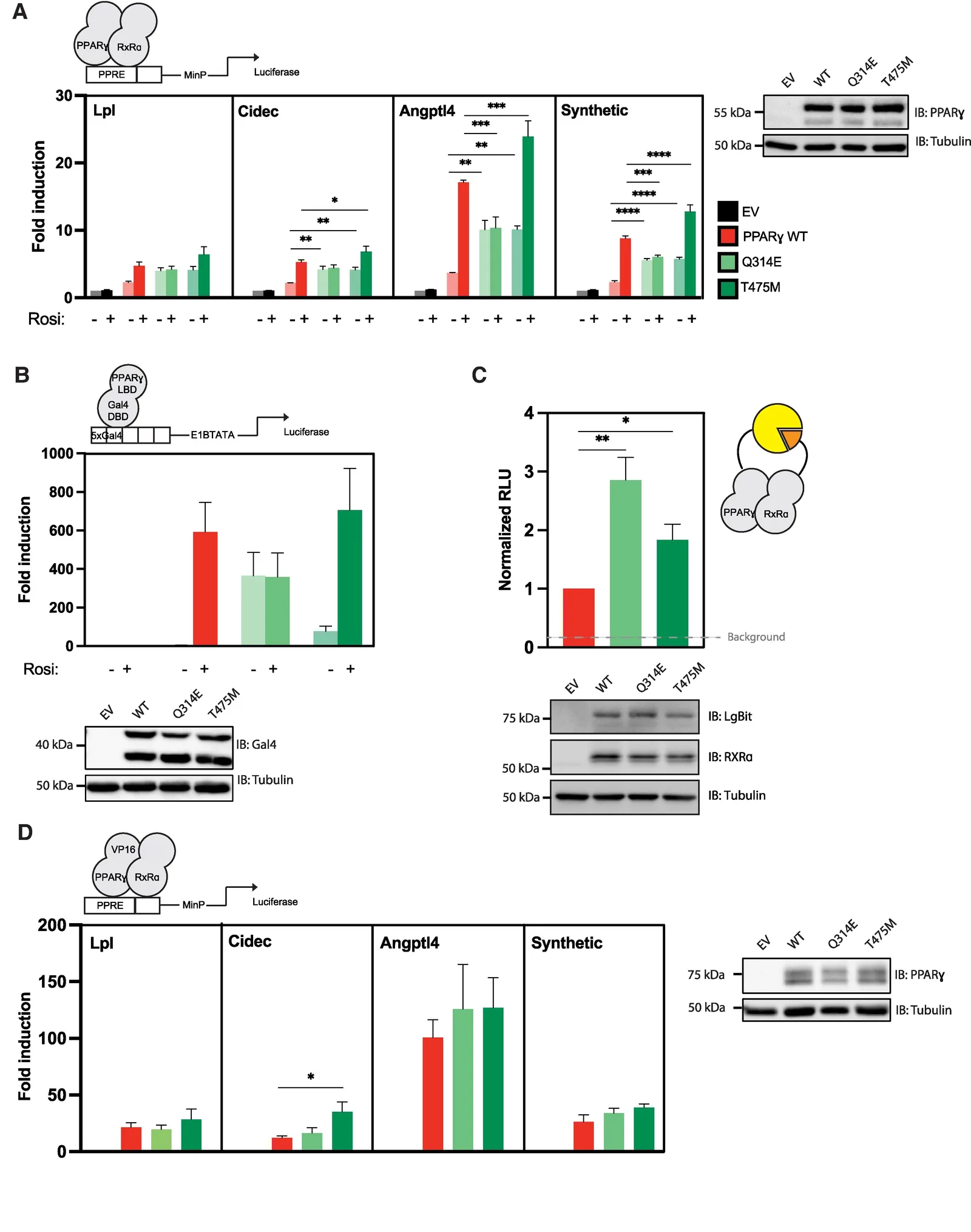
Characterization of luminal muscle-invasive bladder cancer–associated gain-of-function mutations. U2OS cells were transiently cotransfected with expression vectors encoding A, peroxisome proliferator–activated receptor γ–wild-type (PPARγ-WT) or loss-of-function (LOF) mutants, or B, Gal4DBD-PPARγ-WT or Gal4DBD-LOF mutants and different reporter constructs or a Gal4 reporter construct as indicated, in the absence or presence of 1-µM rosiglitazone. Activation of the reporter is expressed as fold induction over that with empty vector (control). C, HEK293T cells were cotransfected with expression vectors encoding PPARγ2-LgBiT or LOF mutants, and smBiT-hRxRα. Luciferase activity is expressed relative to PPARγ2-WT. D, U2OS cells were transiently cotransfected with expression vectors encoding. VP16-PPARγ-WT or VP16-LOF mutants and different reporter constructs in the absence of ligand. Activation of the reporter is expressed as fold induction over that with empty vector (control). Protein expression levels were verified by Western blot for all assays with annotated antibodies. All data are presented as mean values + SEM with n = 4 biologically independent experiments. Mutant results were compared to WT with a Dunnett's multiple comparison test: *.01 < *P* < .05; **.001 < *P* < .01; ***.0001 < *P* < .001; ****P < .0001.

Next, we wished to test our reporter-based framework with GOF PPARγ mutants. We selected 2 previously described bladder cancer–associated mutations affecting highly conserved residues in the LBD of PPARγ (Fig. 1B and 1C): the Q314E mutant previously reported to induce constitutive activity in the absence of a ligand (34), and a T475M mutant that exhibits significantly higher transcriptional activity both in the absence and presence of a ligand (9). In the absence of a ligand, the Q314E mutant displayed constitutive activity on all 4 reporter constructs, with a more pronounced observed effect on the *Cidec*, *Angptl4*, and synthetic reporters compared to WT (Fig. 3A). In the presence of a ligand, the activity of this mutant on the *Cidec* reporter was comparable to WT, whereas this was reduced on the *Angptl4* and synthetic reporters. The T475M mutant showed an increased ability to activate the 4 reporters both in the absence and presence of a ligand compared to WT (Fig. 3A). Consistent with its constitutive activity in the full-length reporter assay (see Fig. 3A), the Q314E mutant activated transcription in the absence of a ligand when analyzing the LBD in isolation, suggesting that the mutation stabilizes the LBD in an active conformation (Fig. 3B). Interestingly, this activity was not increased on ligand addition and tended to be slightly reduced compared to WT. The T475M mutant showed intact LBD integrity in the presence of a ligand (see Fig. 3B). Q314E and T475M both exhibited increased capability to heterodimerize with RXRα, indicating gain in binding affinity for its obligate partner (Fig. 3C), in line with previous reports (9, 34). Last, we evaluated the ability of the mutants to bind to DNA sequences and found that both mutants retained the ability to activate transcription from the 4 reporters, indicating preserved DNA binding to these elements (Fig. 3D). On the *Cidec* reporter, the T475M mutant displayed increased ability to activate transcription, which may reflect a stabilized or higher-affinity interaction with this PPRE (see Fig. 3D). Taken together, our reporter-based framework can distinguish distinct phenotypes of GOF PPARγ mutants.

### Peroxisome proliferator–activated receptor γ ligand-binding domain mutations display distinct effects on cofactor interactions

**Figure 4:**
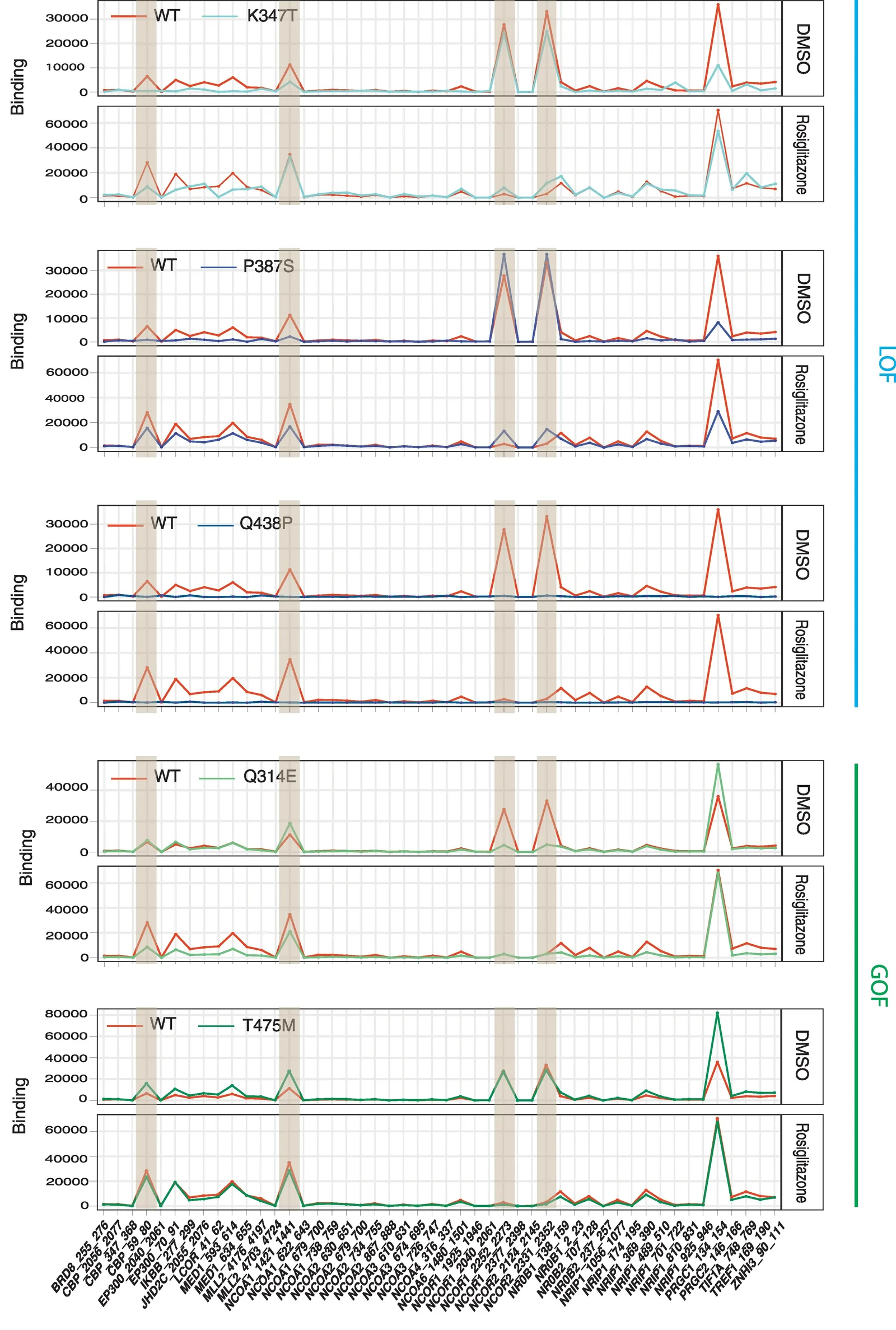
Cofactor profiling of peroxisome proliferator–activated receptor γ (PPARγ) loss-of-function (LOF) and gain-of-function (GOF) mutants. HEK293T cells were transiently transfected with Gal4DBD-PPARγLBD-WT, LOF, or GOF mutants. Cofactor peptide binding in the presence and absence of rosiglitazone is shown for wild-type (WT) (red) and LOF (blue) and GOF (green) mutants. Graph shows quantitative binding for 48 selected peptides and are scaled to strongest binding values. Coactivator peptides CBP 59_80 and NCOA1_1421_1441, and corepressor peptides NCOR1 2252_2273 and NCOR2 2331_2352 are highlighted in taupe.

To validate and expand on these reporter-based findings, we performed NR activity profiling (30). For this, the Gal4DBD-LBD proteins were overexpressed in HEK293T cells and lysates were subsequently tested for quantitative binding to a panel of 101 coregulator peptides; for clarity, 48 peptides are presented in Fig. 4, omitting peptides with very low binding under all conditions, while the full set is provided in Supplementary Fig. S1 (27). WT PPARγ typically showed release of corepressors and robust recruitment of coactivators on ligand binding, as exemplified by the NCoR1 and −2 peptides and CBP and NCoA1 peptides, respectively (see Fig. 4). The 3 PPARγ LOF mutants displayed distinct coregulator binding defects. The K347T mutant exhibited a reduction both in coactivator recruitment and corepressor release on ligand binding (see Fig. 4), suggesting that its reduced transcriptional activity is due to a combination of reduced heterodimerization activity (Fig. 2C) and altered coregulator binding (see Fig. 4).The P387S mutant, which displayed intact heterodimerization and DNA binding (see Fig. 2), showed strongly enhanced binding of corepressors in the absence of ligand and retained corepressor binding in the presence of a ligand. Interestingly, this mutant also displayed strongly enhanced binding of coactivators compared to WT, suggesting an overall increased affinity for coregulators (see Fig. 4). In contrast, and in agreement with the dramatic overall defect observed for this mutant (see Fig. 2), the Q438P mutant completely failed to bind coregulator peptides in the absence or presence of a ligand, suggesting an LBD folding defect that broadly disrupts LBD structure and thus coregulator recruitment. Notably, the PPARγ GOF mutants showed different coregulator binding profiles. The Q314E mutant lacked corepressor binding in the absence of a ligand and displayed reduced coactivator recruitment on ligand induction, which is consistent with its behavior in our reporter-based framework (see Fig. 3A and 3B). By contrast, the T475M mutant displayed an overall profile that was comparable to WT, but with enhanced coactivator binding in the absence of a ligand (see Fig. 3A). Thus in the absence of a ligand, the PPARγ GOF mutations are characterized by either a loss of corepressor binding (Q314E), or increased coactivator binding (T475M), which explains their constitutive activity (see Fig. 3A and 3B). Coregulator profiling may not fully explain the strong ligand-dependent activity of the T475M mutant (see Fig. 3A), which may be due to additional molecular mechanisms.

Taken together, the independent coregulator profiling approach supports our reporter-based framework: Altered activity observed in a Gal4DBD reporter setting (see Fig. 2 and 3), which specifically points to changes in ligand-mediated coregulator interactions, is paralleled by altered binding to coregulator peptides (see Fig. 4), where the net balance between corepressor release/retention and coactivator recruitment reflects transcriptional activity in cells. Therefore, we conclude that the reporter-based framework presented here provides a simple and robust method for deep characterization of PPARγ LOF and GOF mutants.

## Discussion

Despite advances in computational prediction of pathogenic missense variants, experimental evidence of their molecular disease mechanism remains limited, particularly in clinically relevant contexts. Here, we present a simple and robust reporter-based framework that provides a fast and accessible approach for functional characterization of PPARγ mutations. By systematically assessing transcriptional activity, LBD integrity, heterodimerization with RXRα, and DNA binding, our framework enables the deep characterization of mechanistic alterations without the requirement of highly specialized laboratory infrastructure.

As proof of principle, we analyzed 3 previously uncharacterized FPLD3-associated PPARγ variants (32, 35), alongside 2 previously described GOF variants found in luminal bladder cancer (9, 34). Overall, our framework showed each mutant to exhibit distinct alterations in transcriptional activity, LBD integrity, RXRα heterodimerization, and DNA binding. Several lines of evidence support and validate our findings. First, all 3 LOF mutations showed diminished transcriptional activation, which aligns with the established notion in the literature that FPLD3-associated variants invariably destabilize the active transcriptional complex (36). Second, when subjecting these 3 mutants to MITER, the functional classification tool for predicting PPARγ variants to cause FPLD3 (31), K347T, P387S, and Q438P were identified as pathogenic with probabilities of 52.9%, 6.2%, and 77.1%, respectively. These probabilities align with the relative severity of the functional impairments observed in our data. Third, in line with previous reports, the 2 GOF mutations showed an opposite functional profile. The Q314E mutant was previously shown to stabilize H12 in an active conformation, resulting in constitutive transcriptional activity and increased RXRα affinity (34), and, similarly, T475M stabilizes H12 in an active conformation, leading to elevated transcriptional activity and increased RXRα affinity (9). Finally, the coregulator profiling performed here both validated and expanded the findings from the reporter-based framework, with reduced or increased coregulator interactions reflecting the activity of the full-length protein and/or LBD, as also reported for other mutants previously (24, 37, 30).

It has been established that the inheritance modes of missense mutations are closely associated with protein structural features: Recessive mutations are predominantly observed in buried regions of protein structures, whereas dominant mutations are significantly enriched at interfaces that are important for intermolecular interactions (38, 39). Furthermore, LOF and GOF mutations have been shown to differ markedly in their effects on protein structure, including their structural location, predicted effect on protein stability, and clustering in 3-dimensional space (40). LOF mutations typically exert the most disruptive effect on protein structure, while stabilizing mutations may give rise to GOF. The LOF and GOF mutations that we characterized here are consistent with this pattern. Q314E and T475M both localize to the AF2 region, where they stabilize H12, thereby explaining their GOF behavior. Concerning the LOF mutations, K347T is positioned on the AF2 coregulator interaction surface where it likely interferes with the orientation of the surrounding helices essential for coregulator binding. Indeed, K347T showed insufficient corepressor release on ligand induction in our assay. P387S resides in a less critical region of the LBD, where the serine likely disturbs local hydrophobic interactions. Consistently, in our assays this mutant maintained full heterodimerization potential and subsequent DNA binding, hence its mild defect in transcriptional activation. Q438P in contrast is buried within the LBD core. The introduction of a proline in an α-helix can be highly destabilizing (41), thereby causing misfolding of the domain and thereby explaining its dramatic functional defect.

Collectively, our findings demonstrate that our method can resolve functional defects across a broad spectrum, consistent with established patterns reported in the literature. We do, however, acknowledge several limitations of the present study. Our method is a targeted approach that focuses on 4 key functional aspects, thereby excluding other protein-intrinsic aspects such as ligand binding and potential posttranslational modifications, but also—and arguably most important—context-dependent aspects like chromatin context and cell type–specific mechanisms. Our characterization of other FPLD3-associated mutants has exemplified in particular the importance of chromatin accessibility in PPARγ action (24), so investigating PPARγ mutants in the context of chromatin on a genome-wide scale presents an important follow-up step. In addition, combinatorial effects of mutations across multiple domains cannot be exclusively disentangled with this framework. Nevertheless, our method proposes a simple and robust first-line strategy for functional testing of PPARγ variants that can be applied to other NRs as well.

## Data Availability

Some or all datasets generated during and/or analyzed during the current study are not publicly available but are available from the corresponding author on reasonable request.

## Abbreviations

*Angptl4*: angiopoietin-related protein 4 gene
CBP: CREB-binding protein
*Cidec*: cell death–inducing DFFA like effector C gene
DBD: DNA-binding domain
FPLD3: familial partial lipodystrophy type 3
GOF: gain of function
H12: helix 12
LBD: ligand-binding domain
LOF: loss of function
*Lpl*: lipoprotein lipase gene
NcoR: nuclear receptor corepressor
NR: nuclear receptor
PPARγ: peroxisome proliferator–activated receptor γ
PPRE: peroxisome proliferator response element
RXR: retinoid X receptor
WT: wild-type
