# Correction to: “A simple and robust reporter-based framework for deep functional characterization of PPARγ mutants”

**DOI:** 10.1210/endocr/bqag094

**Published:** 2026-08-25

In the above-named article by Baak R, Westland D, de Lange E, Houtman R. and Kalkhoven E (*Endocrinology*. 2026; 167 (5); doi: 10.1210/endocr/bqag024), there was an error in the funding information.

In the originally published article, in the funding section, it read, “This work was supported by the Netherlands Organisation for Health Research and Development (ZonMw) programme for translational research and Diabetes Fonds (project No. 95105007).”

In the corrected article, in the funding section, it reads, “This work was supported by the Dutch Organisation for Knowledge and Innovation in Health, Healthcare and Well-being (ZonMw): 09120012010062.”

The article has been corrected online.

The authors regret the error.

doi: 10.1210/endocr/bqag024

